# The respiratory molybdo-selenoprotein formate dehydrogenases of *Escherichia coli *have hydrogen: benzyl viologen oxidoreductase activity

**DOI:** 10.1186/1471-2180-11-173

**Published:** 2011-08-01

**Authors:** Basem Soboh, Constanze Pinske, Martin Kuhns, Mandy Waclawek, Christian Ihling, Karen Trchounian, Armen Trchounian, Andrea Sinz, Gary Sawers

**Affiliations:** 1 Institute for Microbiology, Martin-Luther University Halle-Wittenberg, Kurt-Mothes-Str. 3, 06120 Halle (Saale), Germany; 2 Institute of Pharmacy, Martin-Luther University Halle-Wittenberg, Wolfgang-Langenbeck-Str. 1 06120 Halle (Saale), Germany; 3 Department of Biophysics, Yerevan State University, 1 A. Manoukian Str., Yerevan 0025, Armenia

## Abstract

**Background:**

*Escherichia coli *synthesizes three membrane-bound molybdenum- and selenocysteine-containing formate dehydrogenases, as well as up to four membrane-bound [NiFe]-hydrogenases. Two of the formate dehydrogenases (Fdh-N and Fdh-O) and two of the hydrogenases (Hyd-1 and Hyd-2) have their respective catalytic subunits located in the periplasm and these enzymes have been shown previously to oxidize formate and hydrogen, respectively, and thus function in energy metabolism. Mutants unable to synthesize the [NiFe]-hydrogenases retain a H_2_: benzyl viologen oxidoreductase activity. The aim of this study was to identify the enzyme or enzymes responsible for this activity.

**Results:**

Here we report the identification of a new H_2_: benzyl viologen oxidoreductase enzyme activity in *E. coli *that is independent of the [NiFe]-hydrogenases. This enzyme activity was originally identified after non-denaturing polyacrylamide gel electrophoresis and visualization of hydrogen-oxidizing activity by specific staining. Analysis of a crude extract derived from a variety of *E. coli *mutants unable to synthesize any [NiFe]-hydrogenase-associated enzyme activity revealed that the mutants retained this specific hydrogen-oxidizing activity. Enrichment of this enzyme activity from solubilised membrane fractions of the hydrogenase-negative mutant FTD147 by ion-exchange, hydrophobic interaction and size-exclusion chromatographies followed by mass spectrometric analysis identified the enzymes Fdh-N and Fdh-O. Analysis of defined mutants devoid of selenocysteine biosynthetic capacity or carrying deletions in the genes encoding the catalytic subunits of Fdh-N and Fdh-O demonstrated that both enzymes catalyze hydrogen activation. Fdh-N and Fdh-O can also transfer the electrons derived from oxidation of hydrogen to other redox dyes.

**Conclusions:**

The related respiratory molybdo-selenoproteins Fdh-N and Fdh-O of *Escherichia coli *have hydrogen-oxidizing activity. These findings demonstrate that the energy-conserving selenium- and molybdenum-dependent formate dehydrogenases Fdh-N and Fdh-O exhibit a degree of promiscuity with respect to the electron donor they use and identify a new class of dihydrogen-oxidizing enzyme.

## Background

Hydrogen and formate are electron donors frequently used by anaerobic microorganisms. Metabolism of hydrogen and formate is often highly interlinked in many bacteria that can oxidize both compounds. This is exemplified in the fermentative metabolism of the enterobacterium *Escherichia coli *where up to one third of the carbon from glucose is converted to formate; formate is then disproportionated to H_2 _and CO_2 _[1-3]. Formate can be metabolized by three membrane-associated, molybdo-seleno formate dehydrogenases (Fdh), termed Fdh-H (associated with hydrogen production), Fdh-N (induced in the presence nitrate) and Fdh-O (also detected during aerobic growth). Fdh-H is encoded by the *fdhF *gene and together with one of the four [NiFe]-hydrogenases (Hyd) of *E. coli*, Hyd-3, forms the hydrogen-evolving formate hydrogenlyase (FHL) enzyme complex.

Fdh-N (FdnGHI) and Fdh-O (FdoGHI) are highly related enzymes at both the amino acid sequence and functional levels [1,4]. They are multi-subunit oxidoreductases each comprising a large catalytic subunit (FdnG or FdoG), an electron-transfer subunit (FdnH or FdoH) and a membrane-anchoring subunit (FdnI or FdoI); the latter has a quinone-binding site that allows transfer of electrons derived from formate oxidation into the respiratory chain [4-6]. Both enzymes have their respective active site located on the periplasmic face of the cytoplasmic membrane and they couple formate oxidation to energy conservation. A key feature of all three Fdh enzymes is the presence of selenocysteine, a *bis*-molybdopterin guanine dinucleotide (*bis*-MGD) cofactor and a [4Fe-4S] cluster in their respective catalytic subunit [4,7]. Although the synthesis of the Fdh-N enzyme is induced to maximal levels during growth in the presence of nitrate, the enzyme is also present at lower levels during fermentative growth [1,5,8]. Fdh-O is synthesized constitutively and is present at low levels aerobically, during fermentative growth and nitrate respiration [6,9].

*E. coli *has also the coding capacity to synthesize four membrane-associated, multi-subunit Hyd enzymes, which are termed Hyd-1 through Hyd-4 [2,10]. Hyd-1, Hyd-2 and Hyd-3 have been characterized in detail. Like Fdh-N and Fdh-O, Hyd-1 and Hyd-2 have their active sites located facing the periplasm [11]. Both enzymes oxidize hydrogen and contribute to energy conservation. Due to the fact that hydrogenases catalyze the reversible oxidation of dihydrogen *in vitro*, the activities of all three characterized [NiFe]-hydrogenases of *E. coli *can be determined simultaneously in a single reaction using hydrogen as electron donor and the artificial electron acceptor benzyl viologen (BV) [12,13]. Moreover, the hydrogen-oxidizing activities of Hyd-1 and Hyd-2 can also be visualized after electrophoretic separation under non-denaturing conditions in the presence of detergent [12]. Because of its apparent labile nature the activity of Hyd-3 cannot be visualized after gel electrophoresis.

It was noted many years ago [14] that in non-denaturing polyacrylamide gels a slowly-migrating protein complex with a hydrogen: BV oxidoreductase enzyme activity, apparently unrelated to either Hyd-1 or Hyd-2, could be visualized after electrophoretic separation of membrane fractions derived from *E. coli *grown under anaerobic conditions. In this study, this hydrogenase-independent enzyme activity could be identified as being catalyzed by the highly related Fdh-N and Fdh-O enzymes.

## Results

### Hydrogenase-independent hydrogen: BV oxidoreductase activity in *E. coli *membranes

**Figure 1:**
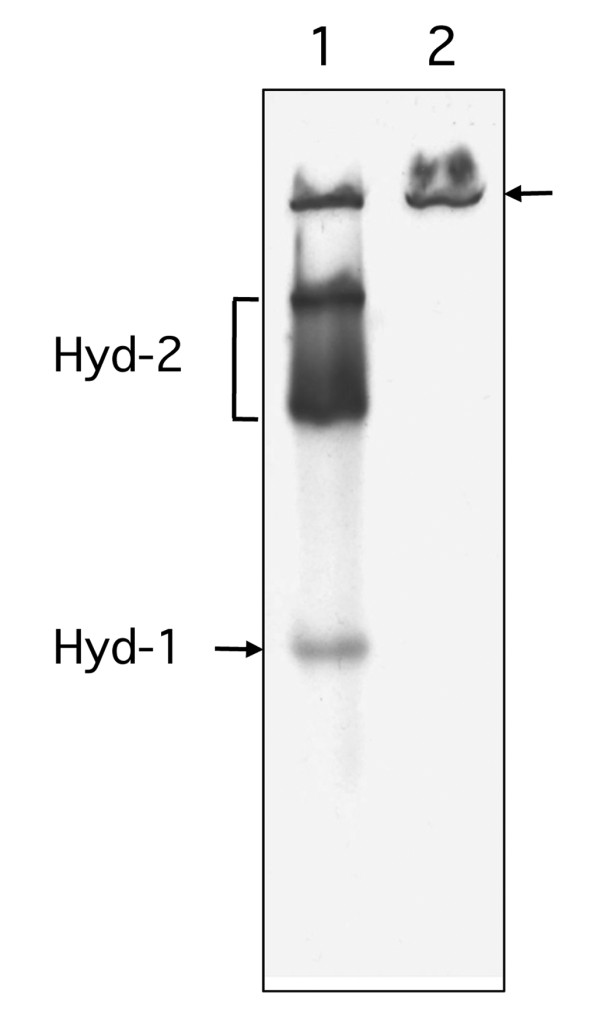
**A *hypF *mutant retains hydrogenase-independent H_2_: BV oxidoreductase activity**. Extracts derived from MC4100 (lane 1) and the isogenic Δ*hypF *mutant DHP-F2 (lane 2) were separated by non-denaturing PAGE and subsequently stained for hydrogenase enzyme activity as described in the Methods section. Strains were grown in TYEP medium with 0.8% (w/v) glucose, pH 6.5. Equivalent amounts of Triton X-100-treated crude extract (50 μg of protein) were applied to each lane. The activity bands corresponding to Hyd-1 and Hyd-2 are indicated, as is the slowly migrating activity band (designated by an arrow) that corresponds to a hydrogenase-independent H_2_:BV oxidoreductase enzyme activity.

Membrane fractions derived from anaerobically cultured wild-type *E. coli *K-12 strains such as P4X [12,15] and MC4100 [16] exhibit a slowly migrating hydrogen: benzyl viologen (BV) oxidoreductase activity that cannot be assigned to either Hyd-1 or Hyd-2. Previous findings based on non-denaturing PAGE [16] estimated a size of approximately 500 kDa for this complex. To demonstrate the hydrogenase-independent nature of this enzyme activity, extracts derived from a *hypF *mutant, which lacks the central hydrogenase maturase HypF and consequently is unable to synthesize active [NiFe]-hydrogenases [17], retained this single slowly migrating species exhibiting hydrogen:BV oxidoreductase activity, while the activity bands corresponding to Hyd-1 and Hyd-2 were no longer visible (Figure 1). This result demonstrates that the activity of this slowly migrating band is completely unrelated to the [NiFe]-hydrogenases Hyd-1, Hyd-2, Hyd-3 or Hyd-4. Note that no active, stained bands were observed when this experiment was performed with a nitrogen gas atmosphere (data not shown).

### Formate dehydrogenases N and O catalyze hydrogen:BV oxidoreduction

**Figure 2:**
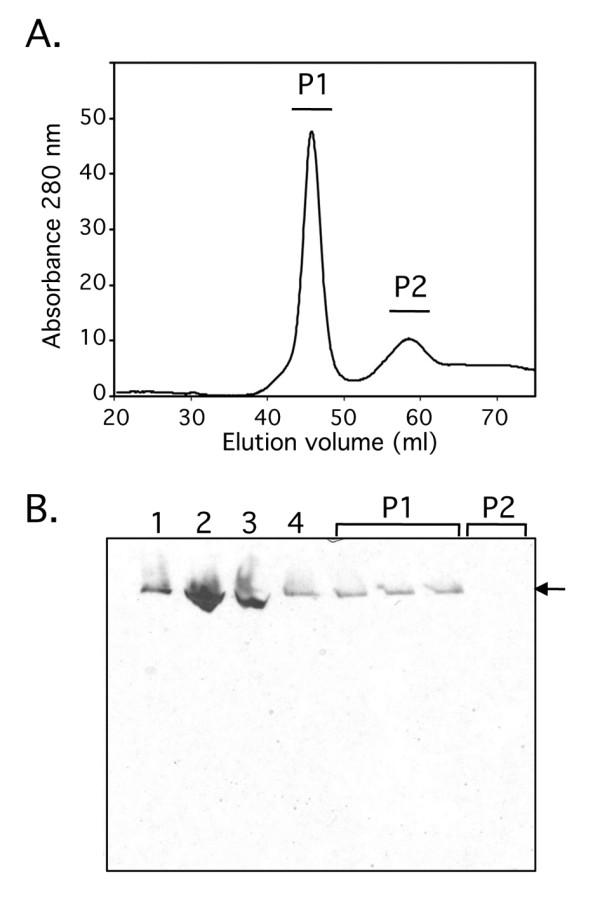
**Chromatographic separation of the H_2_: BV oxidoreductase activity on a Superdex-S200 column**. A. A representative elution profile of the enriched H_2_: BV oxidoreductase enzyme activity after size exclusion chromatography on Superdex-S200 is shown. The absorbance at 280 nm was monitored and the two main elution peaks were labelled P1 and P2. B. Samples of the fractions across the elution peaks P1 and P2 were separated by non-denaturing PAGE and subsequently stained for hydrogenase enzyme activity. Lane 1, crude cell extract (50 μg protein); lane 2, membrane fraction (50 μg protein); lane 3, solubilised membrane fraction (50 μg protein); lane 4, aliquot of the 400 mM fraction from the Q-sepharose column. The arrow identifies the H_2_: BV oxidoreductase enzyme activity.

In order to identify the enzyme(s) responsible for this new hydrogen: BV oxidoreductase activity, the *hypF *deletion mutant was grown anaerobically and the membrane fraction was prepared (see Methods). The hydrogen: BV oxidoreductase activity could be released from the membrane in soluble form by treatment with the detergent Triton X-100. Enrichment of the activity was achieved by separation from contaminating membrane proteins using Q-sepharose anion exchange, phenyl sepharose hydrophobic interaction chromatography and finally gel filtration on a Superdex-200 size exclusion column (see Methods for details). Fractions with enzyme activity were monitored during the enrichment procedure using activity-staining after non-denaturing PAGE. A representative elution profile from the Superdex-200 chromatography step, together with the corresponding activity gel identifying the active enzyme, are shown in Figure 2. Two distinct peaks that absorbed at 280 nm could be separated (Figure 2A) and the hydrogen: BV oxidoreductase activity was found to be exclusively associated with the higher molecular mass symmetric peak labelled P1 (Figure 2B). This peak eluted after 47 ml (V_o _= 45 ml) and was estimated to have a mass of between 500-550 kDa (data not shown).

The band showing hydrogen: BV oxidoreductase activity in Figure 2B was carefully excised and the polypeptides within the fraction were analyzed by mass spectrometry. Both Fdh-O and Fdh-N enzymes were unambiguously identified: the polypeptides FdoG, FdoH, FdoI, FdnG, and FdnH were identified. The catalytic subunits of Fdh-O and Fdh-N share 74% amino acid identity and both enzymes are synthesized at low levels during fermentative growth. Fdh-N is a trimer of trimers (αβγ) with molecular mass of 510 kDa [4] and this correlates well with the estimated size of the protein complex showing H_2_: BV oxidoreductase activity as revealed by non-denaturing PAGE and size exclusion chromatography.

Both Fdh-N and Fdh-O can catalyze the formate-dependent reduction of either BV or DCPIP (2,6-dichlorophenolindophenol) [8,9], whereby Fdh-N transfers electrons much more readily to DCPIP than to BV [8]. Analysis of fraction P1 from the gel filtration experiment revealed a formate: BV oxidoreductase activity of 67 mU mg protein^-1 ^and a formate: DCPIP oxidoreductase activity of 0.64 U mg protein^-1 ^(Table 1). In comparison, the H_2_: BV oxidoreductase activity of fraction P1 was 15 mU mg protein^-1^, while no enzyme activity could be detected when hydrogen gas was replaced with nitrogen gas.

**Table 1:** Activity of enriched enzyme fraction with different electron donors

Electron donor and acceptor^a^	Specific Activity (mU mg protein^-1^)^b^
H_2 _and benzyl viologen	14.8 ± 2.3
Benzyl viologen without an electron donor	< 0.20
Formate and benzyl viologen	1.24 ± 1.0
Formate and PMS/DCPIP	638.3 ± 69

^a ^The buffer used was 50 mM sodium phosphate pH 7.2; BV was used at a final concentration of 4 mM; formate was added to a final concentration of 18 mM; and PMS/DCPIP were added at final concentrations of 20 μM and 78 μM, respectively.

^b ^The mean and standard deviation (±) of at least three independent experiments are shown.

**Figure 3:**
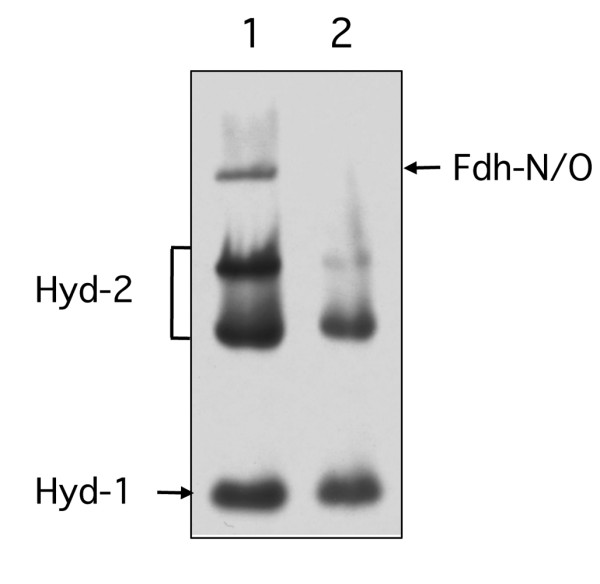
**A *selC *mutant is devoid of the hydrogenase-independent H_2_: BV oxidoreductase activity**. Extracts derived from MC4100 (lane 1) and the isogenic Δ*selC *mutant FM460 (lane 2) were separated by non-denaturing PAGE and subsequently stained for hydrogenase enzyme activity. Equivalent amounts of Triton X-100-treated crude extract (50 μg of protein) were applied to each lane. The activity bands corresponding to Hyd-1 and Hyd-2 are indicated, as is the activity band due to Fdh-N/Fdh-O (designated by an arrow).

All three Fdh enzymes in *E. coli *are selenocysteine-containing proteins [1,2,18]. Therefore, a mutant unable to incorporate selenocysteine co-translationally into the polypeptides should lack this slow-migrating enzyme H_2_-oxidizing activity. Analysis of crude extracts derived from the *selC *mutant FM460, which is unable to synthesize the selenocysteine-inserting tRNA^SEC ^[19], lacked the hydrogenase-independent activity band observed in the wild-type (Figure 3), consistent with the activity being selenium-dependent. Notably Hyd-1 and Hyd-2 both retained activity in the *selC *mutant.

### Fdh-N and Fdh-O can also transfer the electrons from hydrogen to other redox dyes

**Figure 4:**
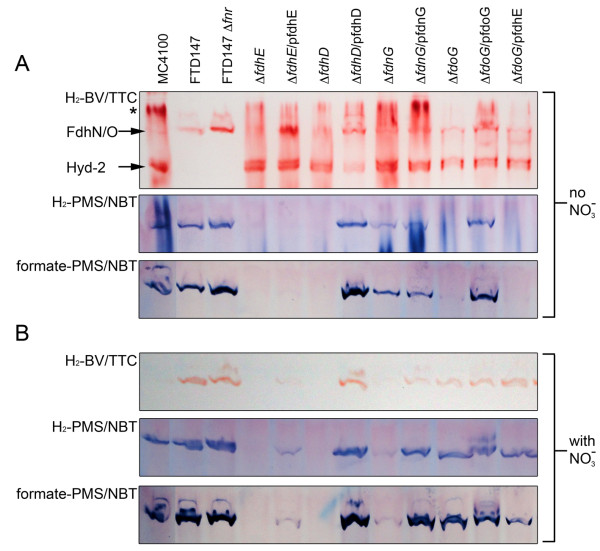
**Analysis of H_2_- and formate-oxidizing activities of Fdh-N/O in different mutant backgrounds**. Small-scale cultures of each strain were grown in TGYEP medium in the absence (A) or presence of nitrate (B). Extracts derived from the strains indicated were separated by non-denaturing PAGE and subsequently stained for H_2_: BV oxidoreductase (top panel), H_2_: PMS/NBT oxidoreductase (middle panel) or formate: PMS/NBT oxidoreductase (bottom panel) enzyme activity as described in the Methods section. Equivalent amounts of Triton X-100-treated crude extract (25 μg of protein) were applied to each lane. The activity band due to Fdh-N/Fdh-O is labelled by an arrow. The activity band due to hydrogenase 2 (Hyd-2) is also labelled in the top panel of part A and was used as a loading control for the experiment. Note the Hyd-2 activity can only be identified as a H_2_: BV oxidoreductase activity. The asterisk indicates hydrogenase activity associated with incompletely solubilised membrane material. The gel stained for H_2_: BV oxidoreductase activity was incubated for 8 h, while the gels stained with PMS/NBT were incubated for 1 h. In the interests of clarity, lanes were labelled based on the key genotype of the strain used. Lanes: MC4100 (wild type); FTD147 (Δ*hyaB *Δ*hybC *Δ*hycE*); FTD147 Δ*fnr *signifies CP1104; Δ*fdhE *signifies JW3862 (Δ*fdhE)*; Δ*fdhE/*pfdhE signifies JW3862 complemented with plasmid pCA24N-*fdhE*^+^; Δ*fdhD *signifies JW3866 (Δ*fdhD*); Δ*fdhD/*pfdhD signifies JW3866 complemented with plasmid pCA24N-*fdhD*^+^; Δ*fdnG *signifies JW1470 (Δ*fdnG*); Δ*fdnG/*pfdnG signifies JW1470 complemented with plasmid pCA24N-*fdnG*^+^; Δ*fdoG *signifies JW3865 (Δ*fdoG*); Δ*fdoG/*pfdoG signifies JW3865 complemented with plasmid pCA24N-*fdoG*^+^; Δ*fdoG/*pfdhE signifies JW3865 complemented with plasmid pCA24N-*fdhE*^+^. Note that BW25113 had an identical phenotype in these experiments to MC4100.

The catalytic subunits of Fdh-N and Fdh-O are encoded by the *fdnG *and *fdoG *genes, respectively [5,6]. To analyse the extent to which Fdh-N and Fdh-O contributed to hydrogen: BV oxidoreductase activity after fermentative growth the activity in mutants with a deletion mutation either in *fdnG *or in *fdoG *was analyzed. Introduction of a deletion mutation in the *fdnG *gene resulted in a slight reduction in intensity of the H_2_: BV oxidoreductase activity band (Figure 4A). The *fdoG *mutation also resulted in a similar phenotype (Figure 4A). Introduction of the *fdnG *or *fdoG *genes on plasmids into the respective mutants restored full activity. An activity band associated with Hyd-2 was used as a loading control for these experiments. Strain FTD147, which has mutations in the genes encoding the catalytic subunits of Hyd-1, Hyd-2 and Hyd-3 [20], and thus cannot synthesize active [NiFe]-hydrogenases, lacked the Hyd-2 activity band but retained the Fdh-N/O hydrogen-oxidizing activity (Figure 4A top panel). Note that the isogenic wild type BW25113 of the JW series of strains had an identical phenoytype to that of MC4100 (data not shown). These experiments demonstrate that under fermentative growth conditions Fdh-N and Fdh-O both contribute to the H_2_: BV oxidoreductase enzyme activity.

Fdh-N and Fdh-O catalyze the formate-dependent reduction of phenazine methosulphate/nitroblue tetrazolium (PMS/NBT), which can be used to visualize Fdh enzyme activity after non-denaturing PAGE [8]. Staining for formate: PMS/NBT oxidoreductase enzyme activity (Figure 4A, bottom panel) revealed that after growth under fermentative conditions, Fdh-O had a stronger contribution to the overall intensity of the activity band than Fdh-N, because while extracts derived from the *fdnG *mutant had similar activity to the wild type, the activity in an extract from the *fdoG *mutant was considerably reduced. Introduction of the *fdoG *gene on a plasmid, however, restored the activity to the mutant (Figure 4A bottom panel). Notably, replacing formate with hydrogen as electron donor revealed that both enzymes also catalyzed the hydrogen-dependent reduction of PMS/NBT (Figure 4A, middle panel). A similar pattern for H_2_: PMS/NBT oxidoreductase activity was observed as was seen for formate: PMS/NBT oxidoreductase activity (compare the middle and bottom panels in Figure 4A). Taken together, these findings suggest that Fdh-N is the more effective enzyme at transferring the electrons from H_2 _to BV/TTC than to PMS/NBT. That Fdh-O is nevertheless effective at catalyzing H_2_-dependent BV reduction is shown in the lane containing an extract derived from CP1104 (labelled FTD147Δ*fnr *in Figure 4) in which an *fnr *mutation was introduced into the hydrogenase-negative strain FTD147 (Figure 4, top panel). Synthesis of Fdh-N is absolutely dependent on the redox regulator FNR [1,21] and thus is absent in an *fnr *mutant. In contrast, Fdh-O activity is apparently up-regulated in the *fnr *mutant (Figure 4A).

### Fdh-N/O show H_2_: BV and H_2_: PMS/NBT oxidoreductase activities in extracts after respiratory growth with nitrate

Biosynthesis of Fdh-N is enhanced when *E. coli *is grown anaerobically in the presence of nitrate [1,5,21], while synthesis of Fdh-O is essentially constitutive [9]. The same strains analyzed in Figure 4A were grown anaerobically in the presence of nitrate and aliquots of crude extracts were separated by non-denaturing PAGE followed by staining for H_2_: BV oxidoreductase, H_2_: PMS/NBT oxidoreductase and formate: PMS/NBT oxidoreductase activities. The gel presented in the top panel of Figure 4B shows clearly a H_2_: BV oxidoreductase activity in extracts of strains FTD147, CP1104 (FTD147Δ*fnr*), as well as in the *fdoG *mutant. The activity in extracts of MC4100 shown in this experiment was only weakly discernable (Figure 4B, top panel, first lane). As anticipated [13], synthesis of Hyd-1 and Hyd-2 was strongly reduced in MC4100 after growth in the presence of nitrate (data not shown). The mutant with a deletion in the *fdnG *gene essentially lacked H_2_: BV oxidoreductase activity but this could be recovered by introduction of the *fdnG *gene on plasmid pCA24N-*fdnG*^+ ^(Figure 4B, top panel).

Aliquots of the same extracts specifically stained to visualize H_2_: PMS/NBT oxidoreductase and formate: PMS/NBT oxidoreductase activities showed a strong Fdh-N-dependent H_2_: PMS/NBT oxidoreductase activity (Figure 4B, middle panel). Substituting hydrogen with formate as electron donor delivered a similar staining pattern (Figure 4B, bottom panel).

### Hydrogen oxidation by Fdh-N and Fdh-O is dependent on the accessory proteins FdhD and FdhE

The *fdoGHI *operon encoding Fdh-O is flanked by *fdhD *and *fdhE*, both of which encode accessory enzymes required for the synthesis of active Fdh enzymes [22,23]. To demonstrate the dependence of the H_2_-oxidizing activities of both Fdhs on FdhD and FdhE, individual mutants lacking either the *fdhD *or the *fdhE *gene were analyzed under the same conditions as described above for the wild type and *fdoG *and *fdnG *mutants. All three activities were absolutely dependent on both FdhD and FdhE (Figure 4). Complementation experiments revealed that while FdhD on a plasmid fully complemented the *fdhD *mutation, plasmid-encoded FdhE only partially complemented the *fdhE *mutation.

## Discussion

We demonstrate here that both of the respiratory formate dehydrogenases Fdh-N and Fdh-O have hydrogen-oxidizing enzyme activity. Together with the three characterized [NiFe]-hydrogenases, these are the only two enzymes in *E. coli *crude extracts that had this activity. These results suggest that the Fdh-N and Fdh-O enzymes show a degree of non-specificity with regard to the electron donor they can use. Notably, formate and dihydrogen (CO_2_/formate, E_o_' = -432 mV [24]) and (H^+^/hydrogen, E_o_' = -414 mV) are both strong reductants.

Previous studies have demonstrated that *E. coli *can couple hydrogen oxidation to nitrate reduction and Hyd-1 and Hyd-2 participate in this process [25]. However, attempts to demonstrate significant hydrogen-dependent nitrate reduction in the absence of Hyd-1 and Hyd-2 did not deliver reproducible levels of hydrogen oxidation, presumably due to the limited hydrogen-oxidizing activity of Fdh-N and Fdh-O. Nevertheless, the findings reported here might have physiological relevance in other microorganisms. For example, enzymes with subunits orthologous to FdnG are found in the obligate dehalorespiring and hydrogen-oxidizing *Dehalococcoides *spp., e.g. strain CBDB1, and have an associated subunit with similarity to hydrogenase membrane-anchoring subunits [26]. Rather than having a selenocysteinyl residue in their presumptive active site they have a seryl residue. It is established that in *E. coli *replacement of selenocysteine with serine abolishes the formate-oxidizing activity of Fdh-H [27]. Moreover, it is also clear that *Dehalococcoides *strain CBDB1 cannot use formate as a substrate, suggesting that this formate dehydrogenase-like enzyme might have another function. One possibility based on the findings presented here might be that it accepts H_2 _as substrate.

As both Fdh enzymes are selenium-dependent, impaired co-translational insertion of selenocysteine prevented synthesis of either enzyme and concomitantly abolished the [NiFe]-hydrogenase-independent H_2_: BV oxidoreductase activity. Moreover, because both Fdh-N and Fdh-O are absolutely dependent on the FdhE and FdhD proteins for activity of the enzyme [22,23], deletion of *fdhE *and *fdhD *also abolished specifically this H_2_: BV oxidoreductase activity. The precise functions of FdhD and FdhE in formate dehydrogenase biosynthesis remain to be established; however, it is likely that they perform a function in post-translational maturation of the enzymes [22].

While it is established that the iron-molybdenum cofactor in nitrogenase catalyzes unidirectional proton reduction as an inevitable consequence of nitrogen reduction [28], the studies here present the first report of a seleno-molybdenum enzyme catalyzing dihydrogen activation. Recent studies have shown that high-valence (oxidation state VI) oxo-molybdenum model complexes can activate dihydrogen at high temperature and H_2 _pressure [29]. The crystal structure of Fdh-N [4] also reveals a similar geometry of the molybdenum atom to these model complexes; however, along with the four *cis *thiolate groups, which are derived from the two MGD cofactors, a hydroxyl from a water molecule and the selenate group from selenocysteine coordinate the Mo atom. The coordination geometry might play an important role in conferring hydrogen activation capability, as the molybdoenzyme nitrate reductase from *E. coli *[30] cannot oxidize dihydrogen. Instead of the selenate ligand, nitrate reductase has an oxo ligand to the Mo, which is contributed by an aspartate residue. In this regard, however, it should be noted that although the third formate dehydrogenase Fdh-H also has similar active site geometry to Fdh-N [4,7], we could not detect a dihydrogen-activating activity associated with this enzyme in our gel system. In contrast to other molybdopterin-containing molybdoenzymes catalyzing oxo-transfer of the oxygen from H_2_O to the substrate, Fdh-H, and presumably also Fdh-N and Fdh-O, catalyze the direct release of CO_2 _and not bicarbonate from formate [31]. The transfer of the proton from formate to a histidine and concomitant reduction of Mo(VI) to Mo(IV) facilitates direct release of CO_2 _with the cofactor returning to the oxidized Mo(VI) state after electron transfer to the iron-sulfur cluster [31]. Such a dehydrogenation reaction could explain the inefficient oxidation of H_2 _by Fdh-N/O demonstrated here. Future studies will focus on testing this hypothesis to characterize the mechanism of dihydrogen activation.

## Conclusions

The energy-conserving formate dehydrogenases of *E. coli *can use dihydrogen as an enzyme substrate. Apart from the [NiFe]-hydrogenases, these enzymes were the only ones in extracts of anaerobically grown *E. coli *that could oxidize hydrogen and transfer the electrons to benzyl viologen or phenazine methosulfate/nitroblue tetrazolium. While the possible significance of this activity to the general anaerobic physiology of *E. coli *remains to be established, this finding has potentially important implications for our understanding of the hydrogen metabolism of other anaerobic microorganisms.

## Methods

### Strains, plasmids and growth conditions

All bacterial strains and plasmids used in this study are listed in Table 2. Bacterial growth of all *E. coli *strains was performed at 37°C. *E*. *coli *cells were cultivated anaerobically in buffered TYEP medium [32] supplemented with 0.8% (w/v) glucose. Where indicated formate was added to a final concentration of 15 mM and nitrate to 15 mM. Aerobic cultures were grown in flasks filled maximally to 10% of their volume, while anaerobic cultures were grown in stoppered bottles filled to the top with medium. When required, kanamycin was added to a final concentration of 50 μg/ml and chloramphenicol to a final concentration 15 μg/ml. Cultures were harvested after reaching an optical density at 600 nm of 0.9 was attained. Cells were collected by centrifugation at 50,000 xg for 20 min at 4°C. Harvested cell pellets were suspended in 50 ml 50 mM MOPS pH 7.5 and re-centrifuged under the same conditions. Washed cell pellets were either used immediately or stored at -20°C until use.

**Table 2:** Strains and plasmids used in this study

Strains	Genotype	Reference or source
MC4100	F^- ^*araD139 *Δ(*argF-lac)U169 ptsF25 deoC1* *relA1 flbB5301 rspL150*^-^	[38]
MC-NG	Like MC4100, but Δ*fdnG*	This work
MC-OG	Like MC4100, but Δ*fdoG*	This work
FM460	Like MC4100, but Δ*selC*	[34]
DHP-F2	Like MC4100, but Δ*hypF*	[17]
FTD147	Like MC4100, but Δ*hyaB*, Δ*hybC*, Δ*hycE*	[19]
CP1104	Like FTD147, but Δ*fnr*	This work
JW1328	BW25113 Δ*fnr*	[39]
JW3862	BW25113 Δ*fdhE*	[39]
JW3866	BW25113 Δ*fdhD*	[39]
JW1470	BW25113 Δ*fdnG*	[39]
JW3865	BW25113 Δ*fdoG*	[39]
Plasmids		

pfdhE	pCA24N *fdhE*^+^	[39]
pfdhD	pCA24N *fdhD*^+^	[39]
pfdnG	pCA24N *fdnG*^+^	[39]
pfdoG	pCA24N *fdoG*^+^	[39]

### Strain construction

Deletions in the *fdnG *and *fdoG *genes were introduced into appropriate strains by P1*_kc _*transduction [33] using strains JW1470 (Δ*fdnG*::Kan^R^) or JW3865 (Δ*fdoG*::Kan^R^) (obtained from the National BioResources Project, Japan) as donors. The *selC *mutation from FM460 [34] was moved in a similar manner into clean genetic backgrounds. Similarly, the *fnr *mutation from JW1328 was transduced into FTD147 to create FTD147Δ*fnr*.

### Measurement of enzyme activity

Hydrogen-dependent reduction of benzyl viologen (referred to as hydrogenase activity) was determined as described [12] using 50 mM sodium phosphate pH 7.2. One unit of enzyme activity is defined as that which reduces 1 μmol of dihydrogen min^-1^. Formate dehydrogenase enzyme activity was assayed spectrophotometrically at RT by monitoring the formate-dependent, PMS-mediated reduction of 2, 6- dichlorophenolindophenol (DCPIP) exactly as described [35] or the formate-dependent reduction of benzyl viologen. The latter assay was performed exactly as for the hydrogenase assay with the exception that 50 mM formate replaced hydrogen as enzyme substrate. One unit of enzyme activity is defined as that which oxidizes 1 μmol of formate min^-1^.

Protein concentration was determined [36] with bovine serum albumin as standard.

### In-gel visualization of enzyme activity

Detection of hydrogenase enzyme activity after non-denaturing PAGE was performed as described [12]. Samples of crude extract or fractions after Q-sepharose, phenyl sepharose and Superdex 200 (5 to 50 μg of protein) were incubated with 4% (v/v) Triton X-100 for 30 min prior to application to the gels. After electrophoretic separation of the proteins, the gels were incubated in 50 mM MOPS pH 7.2 containing 0.5 mM BV and 1 mM 2, 3, 5-triphenyltetrazolium chloride and they were incubated under a hydrogen: nitrogen atmosphere (5% H_2_: 95% N_2_) at room temperature for 8 h. This assay was used to identify the hydrogen-oxidizing activity during the enrichment procedure described below.

Visualization of formate dehydrogenase enzyme activity was performed exactly as described [8] using phenazine methosulfate as mediator and nitroblue tetrazolium as electron acceptor. Visualization of the hydrogen: PMS/NBT oxidoreductase activity associated with Fdh-N and Fdh-O was performed exactly for formate dehydrogenase but formate was replaced by hydrogen gas as enzyme substrate.

### Preparation of cell extracts and enrichment of the hydrogenase-independent hydrogen-oxidizing activity

Unless indicated otherwise, all steps were carried out under anaerobic conditions in a Coy™ anaerobic chamber under a N_2 _atmosphere (95% N_2_: 5% H_2_) and at 4°C. All buffers were boiled, flushed with N_2_, and maintained under a slight overpressure of N_2_.

For routine experiments and enzyme assay determination, washed cells (1 g wet weight) were resuspended in 3 ml of 50 mM MOPS pH 7.5 including 5 μg DNase/ml and 0.2 mM phenylmethylsulfonyl fluoride. Cells were disrupted by sonication (30W power for 5 min with 0.5 sec pulses). Unbroken cell and cell debris were removed by centrifugation for 30 min at 50 000 xg at 4°C and the supernatant (crude extract) was decanted. Small-scale analyses were carried out with 0.1-0.2 g wet weight of cells suspended in a volume of 1 ml MOPS buffer as described above. Cell disruption was done by sonication as described above.

To enrich the protein(s) responsible for the hydrogenase-independent hydrogen-oxidizing activity, crude membranes were isolated from cell extracts routinely prepared from 20 g (wet weight) of cells by ultracentrifugation at 145 000 × g for 2 h. Crude membranes were then suspended in 60 ml of 50 mM MOPS, pH 7.5 (buffer A). Triton X-100 was added to the suspended membrane fraction to a final concentration of 4% (v/v) and the mixture was incubated for 4 h at 4°C with gentle swirling. After centrifugation at 145 000 xg for 1 h to remove insoluble membrane particles, the solubilized membrane proteins present in the supernatant were loaded onto a Q-Sepharose HiLoad column (2.6 x15 cm) equilibrated with buffer A. Unbound protein was washed from the column with 60 ml of buffer A. Protein was eluted from the column with a stepwise NaCl gradient (80 ml each of 0.1 M, 0.2 M, 0.3 M, 0.4 M, 0.5 M and 1 M) in buffer A at a flow rate of 5 ml min^-1^. Activity was recovered in the fractions eluting with 0.4 M NaCl. The fractions containing enzyme activity were brought to a concentration of 0.5 M ammonium sulfate and loaded onto a hydrophobic interaction chromatography column (Phenyl-Sepharose HiLoad; 2.6 × 10 cm) equilibrated with 0.5 M ammonium sulfate in buffer A. Protein was eluted using a stepped ammonium sulfate gradient (60 ml each of 0.4 M, 0.3 M, 0.2 M, 0.1 M and without ammonium sulfate) in buffer A and at a flow rate of 5 ml min^-1^. The hydrogen-oxidizing activity was recovered in the fractions eluting with only buffer A. Fractions containing enzyme activity were concentrated by centrifugation at 7,500 × g in centrifugal filters (Amicon Ultra, 50 K, Millipore, Eschborn, Germany) and applied to a Hi-Load Superdex-200 gel filtration column (2.6 × 60 cm) equilibrated with buffer A containing 0.1 M NaCl. Fractions containing the hydrogen-oxidizing activity eluted after 47 ml (peak maximum); the void volume V_o _of the column was 45 ml and the separation range was from 60-600 kDa. Protein was stored in buffer A containing 0.1 M NaCl at a concentration of 3 mg protein ml^-1^. The activity was stable for several months when stored at -80°C.

### Mass spectrometric identification of proteins

For mass spectrometric analysis the gel band showing H_2_: BV oxidoreductase activity after hydrophobic interaction chromatography was excised and the proteins within the band were *in-gel *digested following standard protocols [37]. Briefly, protein disulfides were reduced with DTT and cysteines were alkylated with iodoacetamide. Digestion was performed at 37°C for two hours using trypsin as protease. ProteaseMax^® ^surfactant was used in the digestion and extraction solutions to improve the recovery of hydrophobic peptides. The peptide extracts were analyzed by LC/MS on an UltiMate Nano-HPLC system (LC Packings/Dionex) coupled to an LTQ-Orbitrap XL mass spectrometer (ThermoFisher Scientific) equipped with a nanoelectrospray ionization source (Proxeon). The samples were loaded onto a trapping column (Acclaim PepMap C18, 300 μm × 5 mm, 5 μm, 100Å, LC Packings) and washed for 15 min with 0.1% trifluoroacetic acid at a flow rate of 30 μl/min. Trapped peptides were eluted using a separation column (Acclaim PepMap C18, 75 μm × 150 mm, 3 μm, 100Å, LC Packings) that had been equilibrated with 100% A (5% acetonitrile, 0.1% formic acid). Peptides were separated with a linear gradient: 0-50% B (80% acetonitrile, 0.1% formic acid) in 90 min, 50-100% B in 1 min, remain at 100% B for 5 min. The column was kept at 30°C and the flow-rate was 300 nl/min. During the duration of the gradient, *online *MS data were acquired in data-dependent MS/MS mode: Each high-resolution full scan (*m/z *300 to 2000, resolution 60,000) in the orbitrap analyzer was followed by five product ion scans (collision-induced dissociation (CID)-MS/MS) in the linear ion trap for the five most intense signals of the full scan mass spectrum (isolation window 2 Th). Both precursor and fragment ions were analyzed in the orbitrap analyzer. Dynamic exclusion (repeat count was 3, exclusion duration 180 s) was enabled to allow detection of less abundant ions. Data analysis was performed using the Proteome Discoverer 1.0 (Thermo Fisher Scientific), MS/MS data of precursor ions in the *m/z *range 350-8000 were searched against the SwissProt Database (version 53.3, taxonomy *E. coli*, 8,852 entries) using Mascot (version 2.2, Matrixscience), mass accuracy was set to 3 ppm and 0.01 Da for precursor and fragment ions, respectively. Carbamidomethylation of cysteines was set as static modification and oxidation of methionine as potential modification. Up to four missed cleavages of trypsin were allowed. Proteins identified by at least two peptides with an expectation value < 0.01 were considered as unambiguously identified.

## Competing interests

The authors declare that they have no competing interests.

## Authors' contributions

BS, MK, MW, CP and KT carried out the biochemical studies. CI performed the mass spectrometric analyses and CI and AS interpreted the data. BS, CP, AT, AS and RGS conceived the study and helped draft the manuscript. RGS wrote the manuscript. All authors have read and approved the manuscript.
